# Upregulation of citrullination pathway: From Autoimmune to Idiopathic Lung Fibrosis

**DOI:** 10.1186/s12931-017-0692-9

**Published:** 2017-12-29

**Authors:** Katerina D. Samara, Athina Trachalaki, Eliza Tsitoura, Anastasios V. Koutsopoulos, Eleni D. Lagoudaki, Ismini Lasithiotaki, George Margaritopoulos, Panagiotis Pantelidis, Eleni Bibaki, Nikolaos M. Siafakas, Nikolaos Tzanakis, Athol U. Wells, Katerina M. Antoniou

**Affiliations:** 1 Department of Thoracic Medicine and Laboratory of Molecular and Cellular Pneumonology, Medical School, University of Crete, Heraklion, Crete Greece; 2 Interstitial Lung Disease Unit, Royal Brompton Hospital, Imperial College, London, SW3 6NP UK; 3 Department of Pathology, Medical School, University of Crete and Heraklion University Hospital, Heraklion, Crete Greece

**Keywords:** Citrullination, Peptidylarginine deiminases, Smoking, Idiopathic pulmonary fibrosis, Rheumatoid arthritis, Interstitial lung disease, Pathogenesis

## Abstract

**Background:**

Increased protein citrullination and peptidylarginine deiminases (PADIs), which catalyze the citrullination process, are central in Rheumatoid arthritis pathogenesis and probably involved in the initial steps towards autoimmunity. Approximately, 10% of RA patients develop clinically significantly ILD. A possible shared role of protein citrullination in rheumatoid arthritis associated interstitial lung disease (RA-ILD), and idiopathic pulmonary fibrosis (IPF) pathogenesis remains unclear.

**Methods:**

We evaluated PADI2 and PADI4 mRNA expression in bronchoalveolar lavage fluid (BALF) cells of 59 patients with IPF, 27 patients RA-ILD and 10 healthy controls. PADI 2 and 4 expression was analyzed by western blot and immunohistochemistry. Citrullinated protein levels were also quantified.

**Results:**

PADI4 mRNA and protein levels were higher in RA-ILD and IPF than controls. Furthermore, PADI4 mRNA levels showed an increase among smokers in RA-ILD. PADI4 expression was detected in granulocytes and macrophages in all groups, with the strongest cytoplasmic expression observed in granulocytes in RA-ILD and IPF. PADI2 mRNA and immunostaining of BAL cells, were similar in all groups among smokers. Overall, stronger staining was observed in current smokers. Citrullinated peptides were significantly increased in IPF compared to RA-ILD and controls. In RA-ILD, protein citrullination strongly correlated with PADI4 expression and anti-citrullinated protein antibodies (ACPAs).

**Conclusions:**

These results suggest that the citrullination pathway is upregulated in IPF and in RA-ILD.

**Electronic supplementary material:**

The online version of this article (10.1186/s12931-017-0692-9) contains supplementary material, which is available to authorized users.

## Background

Rheumatoid arthritis (RA) is an autoimmune disease, characterized by chronic polyarthritis of the small joints and is thought to be driven by antibodies against citrullinated proteins (ACPAs). Citrullination has been suggested to grant an important step in braking immunotolerance or enhancing autoimmune reactivity [1]. ACPAs are positive in about 60–50% of RA patients and may be detected more than 10 years before joint inflammation thus suggesting that RA could be generated outside the joints [2, 3].

Citrullination is a post-translational modification of the amino acid arginine into citrulline. It is a Ca2+ dependent reaction, catalyzed by peptidylarginine deiminases (PADIs) [4]. PADIs are activated by higher concentrations of calcium in cells undergoing apoptosis and during inflammatory responses [5]. Among the known isoforms of PADIs [6], PADI2 and PADI4 have been previously associated with RA pathogenesis [7]. PADI4 polymorphisms leading to higher enzyme levels have been associated with RA susceptibility and HRCT abnormalities in lungs [8]. PADI4 is mainly expressed in macrophages, neutrophils, and eosinophils and triggers neutrophil extracellular traps (NETs) formation [9], whereas PADI2 is highly expressed in macrophages [10, 11]. Citrullinated proteins, most commonly associated with RA are vimentin, filaggrin, cytokeratin and fibrin and have been detected in extra-articular sites [1, 4, 12, 13].

Lungs are the most common site of extra-articular involvement and is associated with autoantibody positivity [14]. Subclinical ILD is identified in 60% of RA patients and clinically significant ILD is present in 10% [15]. UIP is the most common pattern in clinical significant RA-ILD [16], with similar phenotype and mortality as Idiopathic Pulmonary Fibrosis (IPF). Smoking, a known risk factor for both RA and IPF, predispose for lung involvement in RA [14, 17]. Additionally, smoking has been associated with increased concentrations of PADI2 in bronchoalveolar lavage cells [18] and citrullinated proteins are present in the lungs of smokers [19].

Research for the primary pathogenetic events of idiopathic pulmonary fibrosis (IPF) has intensified in recent years, however remain elusive [20]. Indirect evidence of a smoking-related pathogenetic hypothesis exists [21, 22], while citrullination of the lung tissue has already been reported in IPF [23]. The association between smoking and IPF justify the exploration of common pathogenetic pathways linked to smoking, including citrullination. With this background, we assessed the activity of the citrullination pathway via the examination of PADI2 and 4 enzyme expression and protein citrullination levels, in BAL cells, in RA-associated ILD compared to IPF and control subjects.

## Materials and methods

### Patients

Patients were classified as ever smokers and non-smokers. Pulmonary function tests, performed within 1 month of CT, included FEV_1_, FVC, and DL_CO_ corrected for hemoglobin concentration, expressed as percentages of the predicted normal values. The composite physiologic index (CPI) was calculated as 91.0 – (0.65 × percent predicted DL_CO_) – (0.53 × percent predicted FVC) + (0.34 × percentage predicted FEV_1_) [24].

Ninety-six (96) subjects were retrospectively enrolled in this study, consisting of patients with IPF (*n* = 59), patients with RA-ILD (*n* = 27), and healthy control subjects (*n* = 10). 43 IPF patients and 20 RA-ILD patients were being treated at the Interstitial Lung Disease Unit, Royal Brompton Hospital, Imperial College, London, UK and the remaining 16 IPF patients, 7 RA-ILD patients and 10 healthy control subjects were recruited from the Department of Thoracic Medicine, University Hospital of Heraklion, Crete, Greece.

IPF group: The diagnosis of IPF was based on using ATS/ERS clinical and HRCT criteria (*n* = 54) or on open or video-assisted thoracoscopic biopsy, with all biopsies reviewed by the same two histopathologists (*n* = 5), [25]. In accordance with the aforementioned criteria any patient presenting any known cause of pulmonary fibrosis, such as a systemic connective tissue disorder, was excluded from this study using both immunologic screening and rheumatologic clinical evaluation [25]. All IPF patients were newly diagnosed and had not received previous treatment.

RA-ILD group: Criteria for the diagnosis of CTD included the American College of Rheumatology (ACR) 1987 revised criteria for the classification of rheumatoid arthritis (RA) [26]; Patients with RA-ILD had HRCT findings indicative of definite interstitial lung disease, evaluated by two independent assessors. Patients used in our cohort presented with a variety of patterns. As determined by HRCT, RA-ILD was classified as Usual Interstitial Pneumonia-CPFE in twelve (12) out of twenty-seven (27) patients, Fibrotic Nonspecific Interstitial Pneumonia in nine (9), Organizing pneumonia in one (1), Fibrotic NSIP overlapping with Organizing pneumonia in one (1), Diffuse alveolar damage in one (1) and Unclassifiable ILD (bronchiectasis with fibrotic changes, bronchiolitis with fibrotic changes) in three [27].

Control group: The control subjects were patients undergoing bronchoscopy for the investigation of haemoptysis, without any overt pulmonary comorbidities and with normal bronchoscopic findings and cytology results. There were all current or former smokers. Since controls were healthy subjects no pulmonary function test was performed.

Informed consent was obtained from all patients who participated in this study. The study was approved by the Ethics Committees of the University Hospital of Heraklion (IRB number: 1045 and17030) and the Royal Brompton Hospital (REC reference 13/LO/0857).

## Methods

### Pulmonary function tests

All patients were evaluated with complete pulmonary function tests (PFTs), including spirometry, measurement of lung volumes (forced expiratory volume in one second – FEV_1_, forced vital capacity – FVC) and diffusion capacity (DLco). Spirometry, lung volumes using the helium-dilution technique and DLCO (corrected for haemoglobin) using the single breath technique were performed using a computerized system (Jaeger 2.12; MasterLab, Würzburg, Germany). Predicted values were obtained from the standardized lung function testing of the European Coal and Steel Community, Luxembourg (1993).

### BAL fluid processing

BALF was obtained from all patients as previously described [28]. Briefly, a flexible bronchoscope was wedged into a subsegmental bronchus of a predetermined region of interest based on radiographical findings. A BALF technique was performed by instilling a total of 240 mL of normal saline in 60-mL aliquots, each retrieved by low suction. The BALF fractions were pooled and split equally into two samples. One sample was sent to the clinical microbiology and cytology laboratory and the other sample was placed on ice and used for this research. These samples were filtered through 70 nm cell strainers and centrifuged at 400 g for 5 min at 4 °C. Total cell counts were determined.

### RNA extraction:

Total RNA was extracted from 1.5 million cell BALF sample using the TRIzol® reagent (Invitrogen, Carlsband, CA) according to the manufacturer’s instructions. In summary, chloroform is added and the specimens are centrifuged. Total RNA is precipitated from the supernatant with isopropanol, it is washed with 75% ethanol and it is suspended in 50 μl DEPC-treated water. RNA concentration and purity are calculated following measurement of its 260-nm absorbance and 260/280-nm absorbance ratio on a UV spectrophotometer.

### Reverse transcription PCR (RT-PCR):

Reverse transcription reactions for the preparation of first-strand cDNA from 2 μg of total RNA are performed using the AffinityScript™ Multi Temperature cDNA synthesis kit, (Stratagene, La Jolla, CA, USA). Random primers are used as amplification primers.

### Real-time PCR:

Transcript levels of PAD2 & PAD4 were determined using the Mx3000P Real-Time PCR system (Stratagene) and SYBR® Green I Master Mix (Stratagene) according to the manufacturer’s instructions. Primers sequences are shown below:

PADI 2 For: 5′- AAAGGCTTGGGTGGGATGAG-3’.

PADI 2 Rev.: 5′- GGCTCTCGTTGGACAGAATCTT-3’.

PADI 4 For: 5′- CCTGAAGGAGTTTCCCATCAA -3’.

PADI 4 Rev.: 5′- GGTTCCCAAAGGAGTCCAGT -3’.

Actin was used as internal control to normalize *PADI2* and *PADI4* mRNA expression levels, as previously described [28].

### Western blot

1 million BALF cells were lysed in RIPA buffer (R0278-Sigma, Europe) supplemented with protease inhibitor (HALT protease inhibitor, 1,860,932, Thermo Scientific, Europe) and 30 μg total protein were mixed with NuPAGE LDS 4Χ LDS Sample Buffer (Invitrogen Corp., USA) and separated by 12% SDS-polyacrylamide gel electrophoresis. The proteins were then transferred electrophoretically to a nitrocellulose membrane (0.45 μm) (Bio-Rad, Europe). After blocking non-specific epitopes with blotting buffer, membranes were incubated overnight with anti-PADI2 mouse monoclonal antibody (ab-56,928 Abcam-UK) or anti-PADI4 mouse nonoclonal antibody (ab-57,167, Abcam-UK). Horseradish peroxidase-linked anti-mouse immunoglobulin G (IgG) was used as secondary antibody and immunodetection was performed with enhanced chemiluminescence (ECL) Luminata Forte (WBLUF0100-Millipore USA), The mouse anti-actin antibody (MAB 1501, Chemicon, Temecula, CA) was used in order to normalize PADI2 and PADI4 expression. Membranes were visualised with Bio-Rad ChemiDoc XRSplus and semi-quantative analysis by densitometry was performed using the BioRad-Image Lab software.

### Immunocytochemistry

BALF cytospins from four (4) controls, six (6) IPF and five (5) RA-ILD were mounted on charged glass slides and immunohistochemical detection of PADI2 and PADI4 protein expression was assessed using mouse monoclonal primary antibodies anti-PADI2 and anti-PADI4 as described above. Briefly, cytospin slides were incubated with the primary anti-PADI4 and anti PADI2 antibodies, for one hour at room temperature. Negative controls were obtained by omitting primary antibody. Antibody binding was detected by means of the UltraVision Quanto Detection System HRP DAB (Thermo Scientific), without any pre-treatment and according to the manufacturer’s instructions. Colour was developed by 15 min incubation with DAB solution and slides were weakly counterstained with Mayer’s haematoxylin. Immunostaining evaluation was performed by two separate pathologists (AK & EL), assessing the presence of cytoplasmic staining on macrophages, neutrophils and eosinophils.

## Evaluation of protein citrullination

40 μg total protein samples were separated as described above and transferred on to PVDF membranes, followed by citrulline modification using the AMC detection kit reagents (Cat.# 17-347B, Millipore, USA) as described by the manufacturer. The membranes were subsequently analysed as describe above, using the anti-modified citrulline human monoclonal antibody (MABS487, Millipore USA) and secondary HRP-conjugated anti-human antibody included in the AMC detection kit.

## Statistical analysis

Analyses were performed with Graphpad 6.0. Group comparisons were made by analysis of variance, Student *t* test, Wilcoxon rank-sum test, or chi-square testing as appropriate. A *P* value less than 0.05 was considered statistically significant.

PADI2 and 4 mRNA and protein levels results were first evaluated by D’ Agostino-Pearson omnibus normality test. Values reported are median with min and max. Mann-Whitney test was used to examine PADIs expression status among IPF, RA-ILD patient groups and controls.

## Results

Demographic data and PFTs are summarised in Table 1. The three groups were age matched, while the IPF and control group consisted mainly of males, whereas the RA-ILD group consisted more females (12/27).

**Table 1:** Clinicopathological characteristics of all subjects studied

Characteristics	Controls	IPF	RA-ILD	*p* value
Number	10	59	27	
Age	56.6 ± 8.6	64.17 ± 10.41	63.3 ± 10	C vs IPF vs RA-ILD ns*
Gender (male/female)	10/0	45/14	15/12	C vs IPF ns** IPF vs RA-ILD ns p < 0.05 C vs RA-ILD
Non-smokers vs ever-smokers	10/0	36/23	14/13	C vs IPF vs RA-ILD ns**
Py	49.6 ± 8	30.9 ± 21	43.3 ± 43	p < 0.01 C > IPF*** RA-ILD vs IPF vs C ns *P* < 0.01 C > IPF*
FEV1% pred		76.82 ± 17.09	80.9 ± 22	RA-ILD vs IPF*
FVC % pred		73.9 ± 18.08	81.1 ± 24.7	RA-ILD vs IPF ns*
DLco % pred		43.45 ± 13.4	51.9 ± 14.3	P < 0.05 IPF < RA***
CPI (units)		50.69 ± 13.02	42.6 ± 15.5	P < 0.01 IPF > RA*
Macrophages(%)	77.7 ± 13	76.37 ± 11.45	65,1 ± 20.55	C vs RA-ILD vs IPF Ns*
Lymphocytes(%)	18.7 ± 10	9.9 ± 7.9	25.5 ± 20.3	p < 0.001 IPF < RA-ILD*C vs RA vs IPF ns
Polymorphonuclear(%)	1.3 ± 1.5	10.9 ± 11.5	7.8 ± 4.9	C vs IPF vs RA-ILD ns*
Eosinophils (%)	0	4.6 ± 5	2.5 ± 1.8	C vs IPF vs RA-ILD ns*

Values are expressed as means ± standard deviations

*Ordinary one-way ANOVA; *P* < 0.05, following bonferroni adjustment, is considered statistically significant

**χ^2^ test; *P* < 0.05, following Bonferroni adjustment, is considered statistically significant

***Kruskal-Wallis test, P < 0,05, following Dunn’s adjustment is considered statistically significant

Abbreviations: FEV1: forced expiratory volume in one second, FVC: forced vital capacity, DL_CO_: diffusing capacity for carbon monoxide, C: controls, py: packet years

### PADI4 expression in BAL cells

**Fig. 1:**
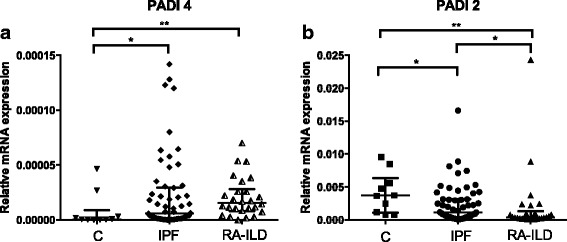
(**a**) PADI4 and (b) PADI2 relative mRNA expression in IPF, RA-ILD and control subjects were analysed by RT-PCR and normalised to actin. * = *p* < 0.05, ** = *p* < 0.01, *** = *p* < 0.001, Mann-Whitney test

PADI4 mRNA transcripts were higher in RA-ILD and IPF compared to controls (*p* = 0.005 and *p* = 0.019) (Fig. 1a and Table 2). In RA-ILD, PADI4 levels tended to be higher than in IPF (median fold change 2.6). PADI4 levels did not vary with age, gender or CPI levels in RA-ILD.

**Table 2:** mRNA expression profiles of PADI2 and PADI4 in IPF, RA-ILD and Control group. Values are expressed as median with min and max. Mann-Whitney test was performed for each pair

	Controls	IPF	RA-ILD	*P*- value
N	10	58	26	
PADI2 (MIN-MAX)	3,67E-03 (7.7E-04 – 9.6E-03)	1.1E-03 (2.6E-05 – 1.7E-02)	3.7E-04 (9.7E-05 – 2.4E-02)	C vs IPF *p* = 0.02* C vs RA *p* = 0.001 IPF vs RA p = 0.02
PADI4 (MIN-MAX)	5.52 E-07 (0–4.7E-05)	6.01E-06 (0–1.4E-04)	1.57E-05 (0–7.0E-05)	C vs IPF *p* = 0.019* C vs RA *p* = 0.0053 IPF vs RA ns
N-smokers	10	36	14	
PADI2 (MIN-MAX)	3,67E-03 (7.7E-04 – 9.6E-03)	1.1E-03 (2.5E-05 – 1.6E-02)	3.9E-04 1.3E-04 – 2.4E-02)	C vs IPF vs RA-ILD ns**
PADI4 (MIN-MAX)	5.52 E-07 (0–4.7E-05)	9.4E-06 (0–1.4E-04)	2.1E-05 (2.8E-06– 7 E-05)	C vs IPF p = 0.019* C vs RA *p* = 0.0032 IPF vs RA ns

*Mann-Whitney test

**Unpaired t-test with Welch correction

In RA-ILD, PADI4 levels were higher in smokers (ex and current) than non-smokers (mean ± SD: 1.3E-05 ± 2.8E-06 vs 2.8E-05 ± 5.9E-06 *p* = 0.035, t-test) and showed a marginal positive correlation with packyears (pearson r:0.59, *p* = 0.082). Since the control group, consisted exclusively of smokers, we performed a second analysis with ever smokers from each disease group and observed that PADI4 levels remained significantly higher in RA-ILD and IPF compared to controls (Kruskal-Wallis test, *p* = 0.005) (Table 2).

**Fig. 2:**
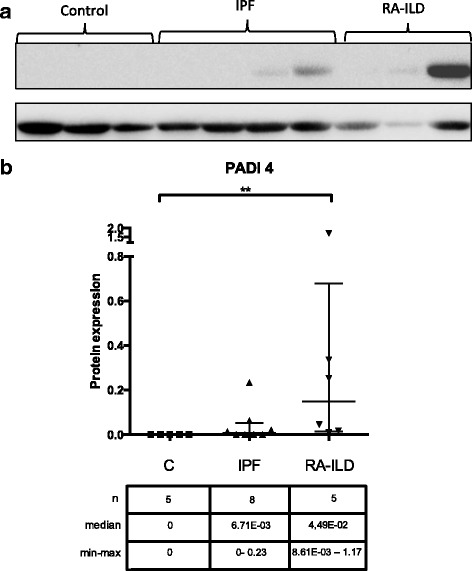
**a**) A representative immunoblot of PADI4 intracellular levels in 3 controls, 4 IPF and 3 RA-ILD subjects. **b**) PADI4 protein expression levels in IPF, RA-ILD and control subjects relative to actin. The bars represent median with range. ** = p < 0.01, Mann-Whitney test

**Fig. 3:**
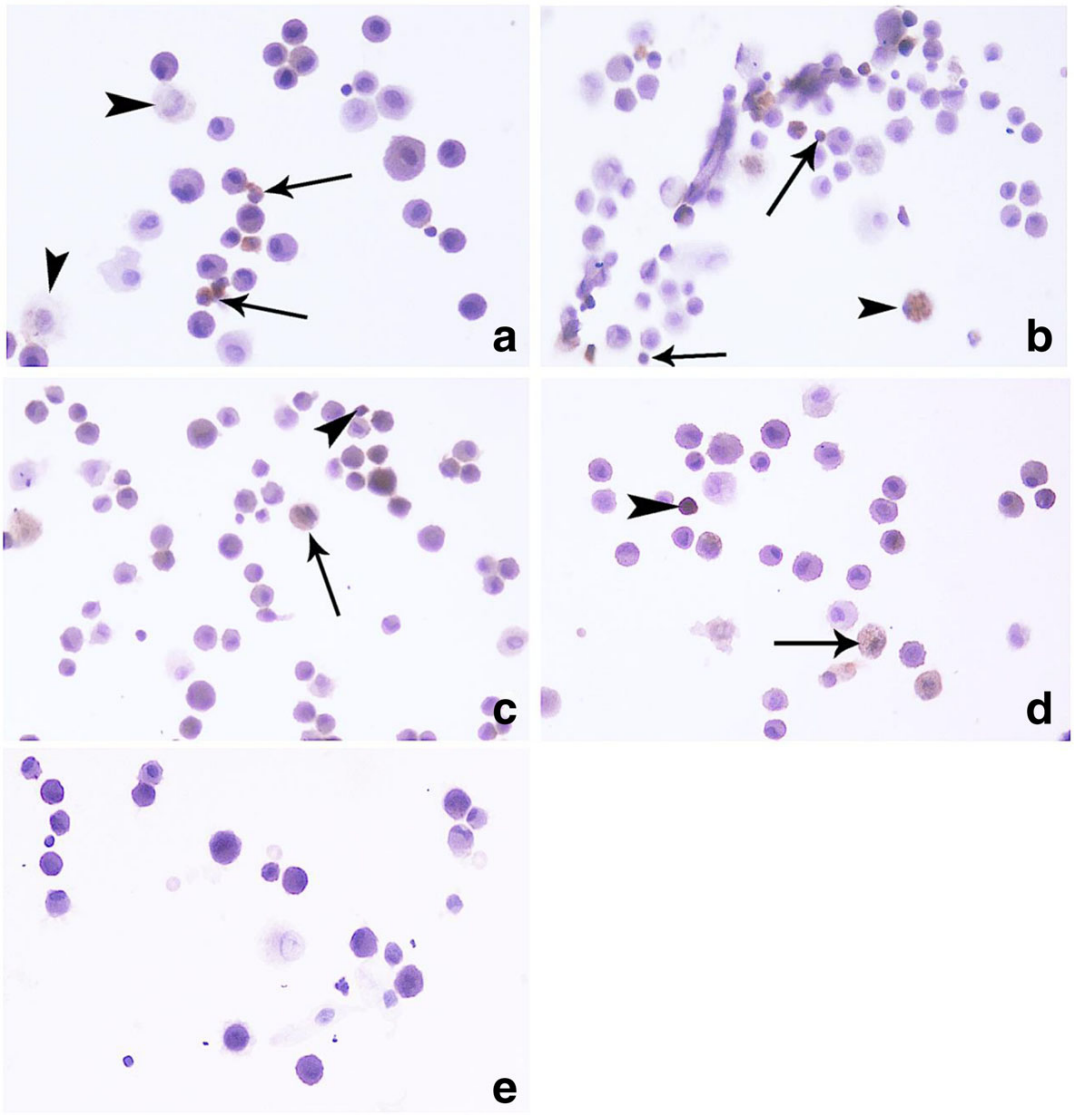
Representative images of BALF cytospins, stained with anti-PADI4 antibody, from IPF (**a**) and RA-ILD (**b**) patients, demonstrated strong cytoplasmic staining mostly in polymorphonuclear leukocytes (arrows) and mild stain in some macrophages (arrowheads), whereas in some IPF (**c**) and control patients (**d**) staining was observed mostly in macrophages (arrows) and only in occasional granulocytes (arrowheads). Negative control (**e**). Magnification X600

Subsequently, we evaluated the protein levels of PADI4 in BAL cells (Fig. 2a and b). PADI4 was significantly higher in RA-ILD compared to controls (Mann-Whitney test *p* = 0.0043), and marginally elevated compared to IPF (*p* = 0.051). Notably, 50% (4/8) of IPF patients, and all RA-ILD patients tested had detectable PADI4 protein levels by western blot in contrast to controls. PADI4 immunostaining was mainly observed in neutrophils and eosinophils, with occasional monocyte/macrophage positive cells, in RA-ILD and in a subset of IPF patients (Fig. 3a and b). Controls and a group of IPF exhibited, mainly monocyte/macrophage cytoplasmic staining and less pronounced neutrophil PADI4 expression (Fig. 3c and d).

### PADI2 expression in BAL cells

PADI2 mRNA levels were significantly lower in IPF and RA-ILD compared to controls (*p* = 0.023 and *p* = 0.001 respectively) and in RA-ILD compared with IPF (*p* = 0.018) (Fig. 1b and Table 2), however PADI2 levels were similar in all groups in ever smokers. PADI2 protein levels were detectable, by western blot, in 2/6 RA-ILD and 1/11 IPF patients and not in controls (Additional file 1: Fig. S1). Immunostaining of BAL cells revealed similar expression of PADI2 in all groups albeit, stronger staining was observed in current smokers. (Data not shown).

### Protein Citrullination levels

**Fig. 4:**
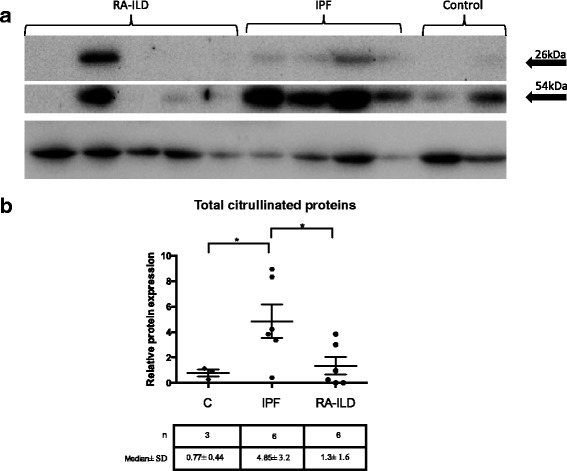
**a**) A representative immunoblot of citrullinated proteins in four (4) IPF, five (5) RA-ILD and two (2) control subjects. **b**) Total citrullinated proteins in IPF, RA-ILD and control subjects relative to actin. The bars represent mean and SD. * = p < 0.05, t-test with Welch correction, kDa: kilodaltons

Given the increased PADI4 expression in RA-ILD and IPF, we subsequently evaluated protein citrullination levels. BAL cells from three (3) control, six (6) IPF and six (6) RA-ILD subjects were analyzed by immunoblotting following chemical modification of citrulline residues. As shown in Fig. 4a, citrullinated proteins were present in all groups. Two main bands were detected, the most prominent had a molecular weight of ~26 kDa and a less prominent of ~54 kDa. Overall, in IPF, citrullinated proteins were higher compared to controls (t-test with Welch correction *p* = 0.03) and RA-ILD (*p* = 0.04) (Fig. 4b).

Notably, although the mean levels of citrullinated peptides were similar in RA-ILD and controls (Fig. 3b) a large variation in protein citrullination was observed in RA-ILD. Further analysis of the RA-ILD group showed that high levels of citrullinated proteins were observed only in ACPA positive subjects (3/5) (pearson *r* = 0.96, *p* = 0.009, *n* = 5). Furthermore, in RA-ILD citrullinated proteins levels positively correlated with PADI4 expression (pearson r = 0.9, p = 0.03, n = 5). In contrast, no correlation of citrullinated proteins and PADI4 expression was observed in IPF or controls.

## Discussion

Idiopathic and autoimmune ILD is associated with ageing and smoking [21, 22, 29]. We hypothesized that the PADIs/citrullination pathway may be a common pathogenetic factor in both IPF and RA-ILD. In the current study, we evaluated the citrullination pathway in the BAL cells of RA-ILD and IPF patients and we identified an upregulation in both groups. Specifically, PADI4 mRNA and protein expression was elevated in RA-ILD and IPF. Pronounced PADI4 expression was observed mainly in neutrophils whilst alveolar macrophages also stained positive with lower intensity. Additionally, protein citrullination was significantly increased in IPF compared to controls. Importantly, we observed that in RA-ILD citrullinated proteins levels positively correlated with PADI4 protein and ACPAs’ levels, in contrast to IPF and controls.

Our results suggest that BAL cells from IPF and RA-ILD patients exhibit increased levels of PADI4 enzyme that was mainly localized in neutrophils. PADI4 is an orchestrator of NETosis, an innate immune response process involving the citroullination of nuclear histones and the release of neutrophil extracellular traps (NETs) in response to pathogen recognition patterns. NETosis may cause local tissue damage and inflammation with already notable roles in chronic and acute lung diseases [30, 31]. NETs are also related to the formation of autoantibodies in various autoimmune diseases [32–34] through exposing citrullinated proteins to the immune system [35] and have already been associated with established RA [36]. Patients with autoimmune associated ILD showed elevated levels of circulating cell- free NETs, together with a decreased DNase activity [37]. A recent report has established that NET formation increases with age and is linked to increased interstitial collagen deposition and pulmonary fibrosis [38]. PADI4 ablation or DNase I administration protected mice against age related organ fibrosis, including pulmonary fibrosis [38]. In IPF, increased number of BAL neutrophils is a predictor of early death [39] and is linked to the morphologic extent of disease [40]. It could be speculated therefore that increased PADI4 expression observed in our study combined to increased neutrophil infiltration could be involved in the pathogenesis of IPF through the prolonged exposure to NETs.

PADI2 levels have been previously correlated with smoking [18] and our study showed that control smokers had equally elevated mRNA levels of PADI2 as IPF and RA-ILD smokers. Furthermore, RA-ILD smokers showed higher levels of PADI4 mRNA expression suggesting an involvement of smoking in PADI4 expression. Smoking, the strongest common environmental risk factor for the development of chronic lung diseases [22], is associated with the citrullination pathway [4, 12, 13]. Furthermore, recent studies in COPD and lung cancer, where smoking is a central pathogenetic factor, showed increased citrullinated proteins [41, 42] while increased lung tissue citrullination has been observed in Interstitial Pneumonias [19, 23].

In the current study we observed that, in RA-ILD, PADI4 protein levels were upregulated in BAL cells and positively correlated with intracellular citrullination. We further examined the extent of protein citrullination and observed similar levels compared to controls. However, citrullination was present in all ACPAs positive and absent in ACPAs negative subjects. Importantly, we observed a positive correlation between protein citrullination, PADI4 protein and ACPAs’ levels. Protein citrullination has been strongly associated with autoantibody occurrence [1]. Additionally, functional haplotypes of PADI4 enzyme leading to increased PADI4 mRNA stability are strongly associated with RA susceptibility [43]. A current hypothesis in RA pathogenesis suggests that the presence of ACPAs define a distinct clinical RA phenotype [44], in which the lung is the site of disease initiation. ACPA positivity is associated with subclinical interstitial lung abnormalities [14] and high titers of ACPAs are associated with ILD irrespectively of smoking status [45]. Our results provide the first evidence of a link between PADI4 and citrullination levels in BAL cells and ACPA positivity, and is consistent with the notion that the lung is a site of citrullinated autoantigen production. According to our knowledge this is the first study suggesting that PADI4 is upregulated in BAL neutrophils in RA-ILD.

IPF and RA-UIP (the most common pattern of RA-ILD) share a clinical and mechanistic phenotype [27], therefore, we also examined the extent of protein citrullination in IPF patients. We observed a strong upregulation of citrullinated proteins in IPF, suggesting the activation of the citrullination pathway. However, we did not detect a correlation between PADI4 expression and total protein citrullination suggesting that other PADIs may be involved. Intriguingly, the detected citrullinated protein(s) at 54 kDa, previously shown to correspond to enolase, vimentin and/or fibrinogen proteins [19] may suggest a possible link with auto-antibodies against vimentin. Recent findings implicate the presence of anti-vimentin autoantibodies in IPF pathogenesis. Increased anti-vimentin antibodies in the serum and in BALF and the levels were correlated with clinical features of the disease [46]. These observations and other findings provide serological and clinical evidence of immune dysregulation in IPF [41, 45, 47–50], however, evidence of autoimmunity in IPF needs to be interpreted with caution [51], as it can be purely a secondary phenomenon. This question lies beyond the scope of the current study and requires a pleotropic approach to the definition of pathogenesis in future work.

The current report does not lack limitations. Although a large number of patient samples was analysed retrospectively for mRNA levels, relatively small number of samples was available for protein studies. Any possible use of PADI4 as a clinically relevant biomarker or as a therapeutic target requires larger numbers and ideally a prospective cohort.

## Conclusion

The results of the current study suggest that citrullination is an active process in both IPF and the rheumatoid lung, strongly implicating the role of neutrophils. Neutrophil activation and NET formation should thus be further studied as they may provide novel insights in the pathogenesis of both Idiopathic and Autoimmune Interstitial Pulmonary Fibrosis.

## Additional file

Additional file 1: Figure S1. A representative immunoblot of PADI2 in three (3) control, six (6) IPF and six (6) RA-ILD subjects. PADI2 protein correspond to the 72kDA band observed. (DOCX 11 kb)

## Abbreviations

ACPA: Anti- Citrullinated Peptide Antibody
BALF: Bronchoalveolar Lavage Fluid
COPD: Chronic Obstructive Pulmonary Disease
CPI: Composite Physiologic Index
DL_CO_: Diffusing capacity for carbon monoxide
FEV_1_: Forced expiratory volume in one second
FVC: Forced vital capacity,
HRCT: High Resolution Computed Tomography
IPF: Idiopathic Pulmonary Fibrosis
NS: Not significant
PADI: Peptidylarginine deiminase
RA: Rheumatoid Arthritis
RA-ILD: Rheumatoid Arthritis – Interstitial Lung Disease
