# Supplementary material for: Upregulation of citrullination pathway: From Autoimmune to Idiopathic Lung Fibrosis
Source: Respir Res. 2017 Dec 29;18:218. doi: 10.1186/s12931-017-0692-9 (PMC5747943; doi:10.1186/s12931-017-0692-9)
Supplement: Additional file 1: Figure S1. — A representative immunoblot of PADI2 in three (3) control, six (6) IPF and six (6) RA-ILD subjects. PADI2 protein correspond to the 72kDA band observed. (DOCX 11 kb) [file 12931_2017_692_MOESM1_ESM.docx]

Figure Legend

**Supplementary figure 1.**

A representative immunoblot of PADI2 in three (3) control, six (6) IPF and six (6) RA-ILD subjects. PADI2 protein correspond to the 72kDA band observed.
